# Return to work among self-employed breast cancer survivors from the CANTO cohort

**DOI:** 10.1038/s41598-026-41157-3

**Published:** 2026-03-12

**Authors:** Chloé Lopez, Idlir Licaj, Agnès Dumas, Julie Havas, Antonio Di Meglio, Ines Vaz Luis, Barbara Pistilli, Fabrice André, Anne-Laure Martin, Dominique Delmas, Christelle Jouannaud, Courèche Kaderbhai, François Cherifi, Mario Campone, Marie-Ange Mouret-Reynier, Garazi Ruiz-de-Azua, Gwenn Menvielle

**Affiliations:** 1 Université Paris-Saclay, UVSQ, Gustave Roussy, Inserm, CESP, 94800 Villejuif, France; 2 SESSTIM (Economic and Social Sciences of Health and Medical Information Processing), INSERM, Aix Marseille Univ, IRD, ISSPAM, CALIPSO Team, Marseille, France; 3 Interdisciplinary Department for the Organization of Patient Pathways (DIOPP), 94800 Villejuif, France; 4 Medical Oncology Department, Gustave Roussy, 94800 Villejuif, France; 5 Data and Partnerships Department, Unicancer, Paris, France; 6 Medical Oncology Department, Institut Godinot, Reims, France; 7 Medical Oncology Department, Centre Georges-François Leclerc, Dijon, France; 8 Medical Oncology Department, Centre François Baclesse, Caen, France; 9 Medical Oncology Department, Institut de Cancérologie de l’Ouest-Nantes, Saint-Herblain, France; 10 Medical Oncology Department, Centre Jean Perrin, Clermont-Ferrand, France

**Keywords:** Cancer, Diseases, Health care, Medical research, Oncology

## Abstract

The aim of this study was to assess return to work (RTW) after breast cancer (BC) by baseline employment status. Using data from the CANTO cohort (NCT01993498), we examined RTW among women below age 57 at BC diagnosis 2 and 4 years after BC diagnosis. We also assessed continuous work between years 2 and 4 post-diagnosis. Poisson regression models investigated associations between baseline employment (self-employed vs. employees) and RTW outcomes, adjusting for clinical, sociodemographic, and work-related factors and quality of life. We used education (above high school; high school or lower) as a proxy to further distinguish between white collar self-employees vs. blue collar self-employees (vs. employees). About 8% of women were self-employed at diagnosis. RTW was slightly higher among the self-employed compared to employees 2 years (*N* = 3178; PR = 1.04, 95%CI 0.98–1.10) and 4 years after diagnosis (*N* = 2329; PR = 1.05, 0.99–1.12). This effect was limited to white collar self-employed women. Among those who returned to work 2 years after diagnosis (*N* = 1683), self-employed women showed a 1.15 (1.01–1.31) higher prevalence of continuous work until year 4 post-diagnosis than employees. Self-employed women do not strongly differ from employees when assessing RTW at specific time points, however once returned to work, they experience fewer work interruptions than employees. This was observed in adjusted models, showing the intrinsic importance of job status in the RTW process. These findings highlight the importance of viewing RTW as an ongoing process, in order to provide appropriate and sustained support.

**Supplementary Information:**

The online version contains supplementary material available at 10.1038/s41598-026-41157-3.

## Introduction

Work cessation during breast cancer (BC) treatment is common and return to work (RTW) after this is often considered an important milestone towards recovery and normalcy^1^. However, many women struggle to reintegrate the labor market after BC. Employment rates one year after diagnosis vary from 54% in France to 82% in the United States and are lower than among the general population^2–5^.

Several clinical and social factors affect RTW of BC survivors (BCS). Advanced stage at diagnosis, treatments received and their impact such as fatigue, chronic pain, and anxiety decrease RTW^6^. A younger age, the presence of economically dependent children and a higher socioeconomic position are associated with increased RTW^6–10^. Meanwhile, the association between having a partner and RTW is inconclusive^7,10^. Having physically or psychologically demanding work is also linked with decreased RTW^11^.

In addition work contract characteristics, such as being self-employed or an employee, may also influence RTW outcomes. Indeed, self-employed workers have fewer social advantages than employees, in particular in case of sick leave^12^, and can face stronger uncertainty over loss of income. Furthermore, self-employed workers are more likely to prioritize their business over their health^13,14^. At the same time, self-employed workers can face challenges once they return to work after cancer that may not apply to employees, due to job responsibilities and lack of social and legal support^15,16^.

However, most of the existing literature investigating work contract characteristics in relation to RTW combined both sexes, predominantly including studies that are not BC specific^4,12,15^. Few studies have focused on BCS but they were based on reduced samples. These studies reported contrasted findings, with either no difference in RTW or higher RTW rates among self-employed workers compared to employees^17–19^. Additionally, while RTW is generally measured at specific time intervals following cancer diagnosis, there is a notable lack in longitudinal studies that map the work trajectory over time. Furthermore, the diversity in job types among self-employed workers ranges from manual jobs to professionals engaging in intellectual jobs, and potentially influences RTW outcomes. Nonetheless, this aspect remains unexplored.

Despite being underrepresented in cancer research^20^, self-employed workers made up 10% of female workers in the European Union and 8% in France in 2018^21^. The share of self-employment is increasing in most European countries as a result of emerging employment models since the early 2000s and various national support initiatives for self-employment^16^. Given the growing attention to the long-term socio-economic impact of BC, understanding how employment status influences RTW trajectories is essential for tailoring supportive interventions. In this context, our main objective was to study the work-related outcomes based on employment status (employee vs. self-employed) following a BC diagnosis, using data from the CANTO cohort. We focused on evaluating the direct effect of employment status on three outcomes: (a) RTW 2 years after BC diagnosis, (b) RTW 4 years after BC diagnosis, and (c) continuous work between years 2 and 4 post-diagnosis after having returned to work 2 years after BC diagnosis among women who were working 2 years after diagnosis.

## Materials and methods

### Data source

The longitudinal and prospective CANcer TOxicity cohort (CANTO; NCT01993498) included women diagnosed with stage I-III BC between 2012 and 2018 from 26 participating cancer centres throughout metropolitan France. Women with stage IV BC, with local or distant BC recurrence, with history of cancer in the 5 years before the study (other than basal cell carcinoma of the skin and cervical carcinoma in situ) or who received BC treatment before the start of the study were excluded from CANTO. More details are available elsewhere^22^.

We used data collected at diagnosis and during follow-up visits 1 year (the visit took place 3–6 months after the end of primary treatment including breast surgery, chemotherapy and/or radiotherapy), 2, 3 and 4 years post-diagnosis. Treatment and classification of tumors were extracted from medical records. The patient’s medical history, pre-diagnostic comorbidities and physical toxicities related to treatment were collected during clinical examinations. Physical and psychological symptoms were collected using validated Patient Reported Outcome Measurements (PROMs). Sociodemographic data (age, family and social situation) and work-related information were collected through self-administered questionnaires at years 2, 3 and after diagnosis.

**Fig. 1:**
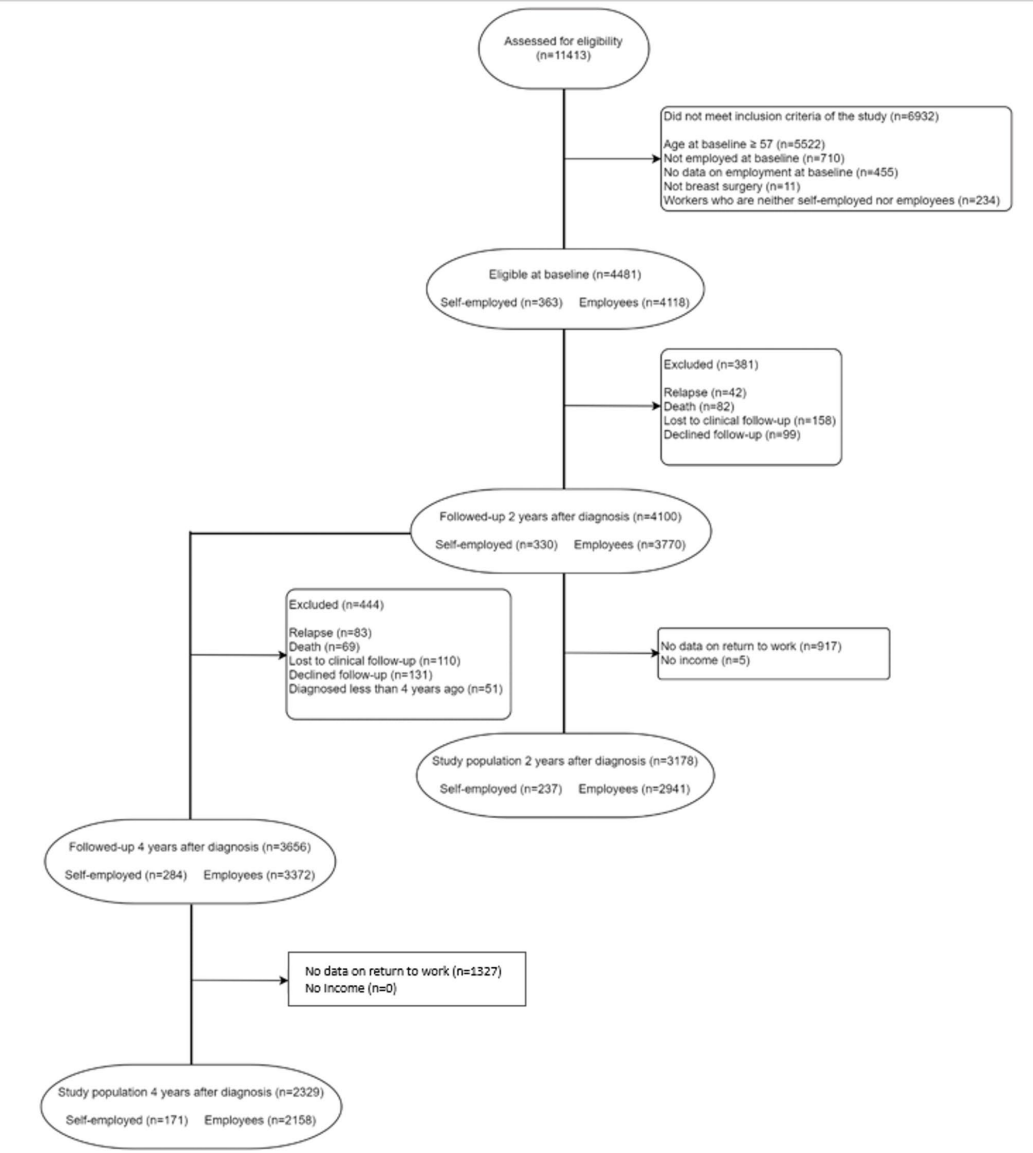

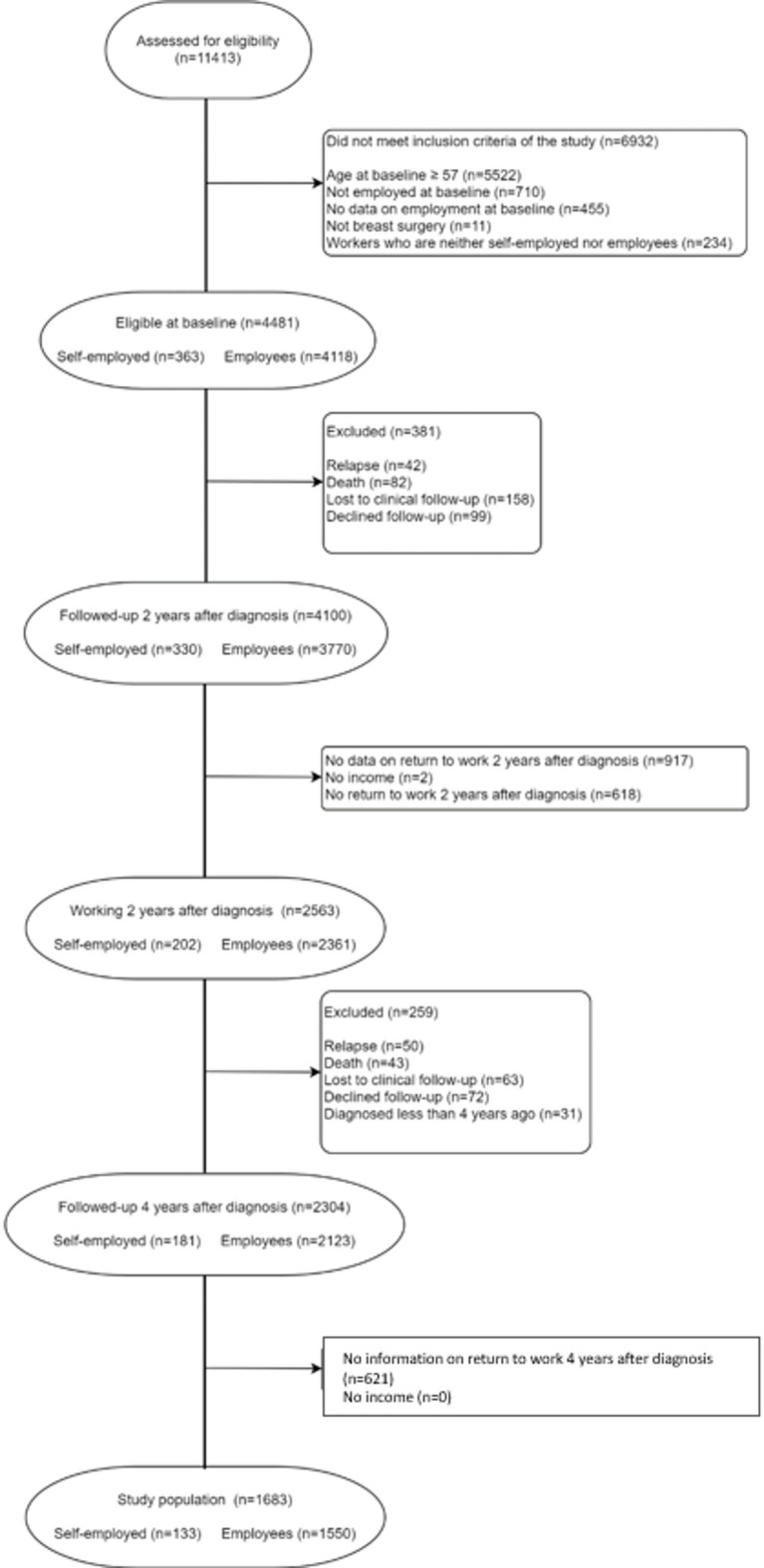
**A** Flow-chart of the 2-year RTW cohort (*n* = 3178) and 4-year RTW cohort (*n* = 2329) after diagnosis. **B** Flow-chart of the continuous work cohort (*n* = 1683). CANTO cohort, France, 2012–2018.

Analyses were conducted in three study populations specific for each study objective: *the 2-year RTW cohort*, to study RTW 2 years after diagnosis; *the 4-year RTW cohort*, to study RTW 4 years after diagnosis; and *the continuous work cohort*, to study continuous work between years 2 and 4 after diagnosis. All study populations were restricted to women below age 57 who were working at BC diagnosis and who received breast surgery. This is because people near retirement age can use legal means to retire instead of returning to work (e.g., 3 year sick leave for individuals with a cancer diagnosis, unemployment benefits, early retirement program), creating selection bias among those who do return. We further excluded women who died, experienced a relapse, decided to exit CANTO or were lost to follow-up before the outcome was measured, as well as women who were not diagnosed long enough ago to reach the time point for the outcome measurement (i.e. 2 years after BC diagnosis for the 2-year RTW cohort, 4 years for the 4-year RTW cohort, and 2 and 4 years for the continuous work cohort). We also excluded women who had no information on the outcome or who reported having no household income. In France, this is highly unusual as a minimum income is available even for individuals without resources. Detailed flow-charts of the study populations are presented in Fig. 1A, B. As shown in Fig. 1A, B, for the 2-year RTW cohort 158 people were lost to follow-up, 917 did not have data on RTW and 5 reported no income. For the 4-year RTW cohort, 110 additional people were lost to follow-up and 1327 additional had no data on RTW. Finally, for the continuous work cohort, 221 women were lost to follow up, 1538 did not have information on RTW and 2 did not have information on income.

### Variables

We first investigated RTW 2 and 4 years after BC diagnosis as a binary variable (yes/no), defined using self-reported information on work status collected at years 2 and 4 post-diagnosis. We then investigated continuous work between years 2 and 4 post-diagnosis among women who returned to work 2 years after diagnosis as a binary variable: had continuous employment over this period vs. experiencing any work discontinuity (having sick leave of at least one month (cumulative, where multiple short sick leaves count toward the cumulative ≥1month threshold), unemployment period, retirement, other (e.g., unpaid leave)). However, to assign an occupational status to participants who reported multiple categories, we applied the following priority order: retirement, sick leave, employment, unemployment, invalidity, and other. For example, if a participant indicated both retirement and employment, they were classified as retired. This outcome was defined using self-reported information on work discontinuity during the past 12 months collected at year 3 and 4 post-diagnosis.

Employment status at diagnosis was categorized into a binary variable: self-employed or employee. A small number of women combined self-employment with an employee’s job and thus potentially benefited of paid sick leave (*n* = 34). These individuals were classified as employees. To further differentiate the employment status, we used a three-category variable: employees, white collar self-employed worker (e.g., liberal profession, company director) and blue-collar self-employed worker (farmer, storekeeper, craftswomen). The distinction was made based on education level where white-collar self-employed workers were proxied with women holding a diploma higher than high school (tertiary education) and blue-collar self-employed workers with women holding a high school diploma or lower. Due to insufficient numbers, we did not conduct a detailed analysis for continuous work between years 2 and 4 post-diagnosis in this subpopulation.

The following covariates were included: clinical variables at diagnosis (stage, comorbidities -measured using the Charlson index^23^-,a binary variable assessing the presence of at least 3 additional comorbid medical conditions not captured by the Charlson index, and treatment), sociodemographic at diagnosis (age, presence of a partner, presence of an economically dependent child, and household income per consumption unit) and work related variables at diagnosis (weekly working hours and work-life imbalance) collected at diagnosis, and quality of life variables (fatigue, distress and physical functioning as continuous variables) collected at year 1 and 2 post-diagnosis for the 2-yeart RTW cohort, and 4-year RTW and continuous work cohorts, respectively. Fatigue and physical functioning were measured using the QLQ-C30 questionnaire, and distress using the HADS questionnaire^24,25^. Categories of each variable are presented in Table 1. Although used as continuous variables in statistical analyses, quality of life variables were described as categorical to improve readability, using the validated thresholds (39 for severe fatigue and noncase [0–14], doubtful [15–22], case [over 23] for distress)^24,25^.

### Statistical analysis

To study how RTW was associated with employment status, we used multivariable Poisson regression models with robust variances to estimate prevalence ratio (PRs)^26^. We tested a random effects Poisson model to account for potential clustering by centre; however, as this did not significantly influence the results, the effect was not retained in the final analyses.

We measured the multi-collinearity between all variables using variance inflation factors (VIF). Since all VIFs were close to 1, no potential collinearity problem was identified. The linearity of the quantitative variables was also checked.

Although main analyses were conducted on women aged 57 and under at the time of diagnosis, we further conducted a sensitivity analysis, repeating all analyses on women under 60 at the time of diagnosis. Although the minimum retirement age was 62 at the time data was collected, individuals could work until age 67. Self-employed might be more likely to work beyond age 62 due to financial needs or lack of employer-sponsored retirement plans. We therefore expect differences in RTW by employment status to be more pronounced among women under 60 compared to women under 57 at diagnosis.

We performed multiple imputations by chained equations to handle missing data, which ranged from 0% to 9.7% (Supplementary Table 1). We created 8 imputed datasets, using exposure, outcomes and confounders, in addition to the following auxiliary variables: level of diploma, importance given to work and PROs at diagnosis and one year after diagnosis). Statistical analyses were performed on the R software version 3.6.3 and R Studio version 4.3.3. The main packages used were epiR, epiDisplay, prettyR, dplyr, geepack, sandwich, lmtest, mice and miceadds. In addition, complete case analyses were performed.

### Ethics approval and consent to participate

CANcer TOxicities is an observational study involving humans, conducted with authorisation from the national regulatory authorities. Specifically, the study received approval from the French National Agency for the Safety of Medicines and Health Products (ANSM): Clinical trial authorisation not involving a health product: n° B111158-20 and Registration number – Research and Biological Collection: ID-RCB:2011-A01095-36. In addition, the study was approved by the Comité de Protection des Personnes (CPP, Committee for the Protection of Individuals): n° 11–039. CPPs are responsible for ensuring the ethical conduct of research in accordance with Article L1123-7 of the French Public Health Code (CSP). This approval was granted by CPP Ile-de-France VII – Hôpital de BICETRE.

All research was performed in accordance with relevant regulations. Informed consent was obtained from all participants involved in the study. This research was conducted in accordance with the Declaration of Helsinki. We confirm that CANTO has all required permissions to use the validated questionnaires included in this study.

## Results

Analyses for RTW 2 years after BC diagnosis (the 2-year RTW cohort) included 2941 employees and 237 self-employed women, analyses for RTW 4 years after BC diagnosis (the 4-year RTW cohort) included 2158 employees and 171 self-employed women, and analyses for continuous work between year 2 and year 4 post-diagnosis (the continuous work cohort) included 1550 employees and 133 self-employed women.

Characteristics of the three study populations are presented in Table 1. About 8%, 7% and 9% of the women were self-employed in the 2-year RTW cohort, 4-year cohort and continuous work cohort, respectively. In the three populations, self-employed women were older and placed higher importance on their professional life at diagnosis (e.g. for the 2-year RTW cohort, used to analyze RTW 2 years after diagnosis, 19% for self-employed vs. 9% for employees). In addition, in the 2-year RTW cohort, the self-employed were less likely to receive chemotherapy (58% versus 64%) than the employees and reported less severe fatigue one year after diagnosis (34% versus 42%), differences that were not observed in the 4-year RTW cohort or the continuous work cohort. Other characteristics showed no significant differences between the self-employed and employees.

As a comparison, supplementary Table 2 shows the characteristics of women who died, experienced a relapse, decided to exit CANTO or were lost to follow-up before the outcome was measured. They were younger than the overall cohort, in particular among the self-employed. They had a more advanced stage at diagnosis, and received chemotherapy or hormonotherapy less frequently. In addition, for the population followed-up 4 years after BC diagnosis, they were more likely to have mastectomy and sentinel dissection (the latter only among employees). Supplementary Table 3 shows information on women who did not answer RTW questions. They were younger, had a more advanced stage at diagnosis and had a higher Charlson index.

Two years after diagnosis, 85% of the self-employed and 80% of employees had returned to work (Table 2). The univariable analysis showed higher RTW among the self-employed compared to employees (PR = 1.06, 95% CI = 1.01–1.12) and similar results were observed in the multivariable analysis (PR = 1.04, 0.98–1.10). Return to work 4 years after diagnosis, was 86% for the self-employed compared to 81% for the employees. The multivariable analysis showed slightly higher RTW among the self-employed compared to employees (PR = 1.05, 0.99–1.12).

When differentiating type of self-employed women, 89% of white-collar workers had returned to work two years after diagnosis compared with 79% of blue-collar workers. Four years after diagnosis, the corresponding figures were 90% for white-collar and 79% for blue-collar workers. The multivariable analysis showed a higher prevalence of RTW both 2 and 4 years after diagnosis among white-collar self-employed workers (PR = 1.05, 1.00-1.12 and PR = 1.06, 0.99–1.13 for RTW 2 and 4 years after diagnosis, respectively) compared to employees, but no difference was observed between blue-collar self-employees and employees (PR = 1.00, 0.90–1.11 and PR = 1.04, 0.92–1.18, respectively).

Among women who returned to work 2 years after BC diagnosis, 67% of self-employed women worked continuously until year 4 post-diagnosis compared with 57% among employees. Compared to employees, self-employed women had a 1.15 (1.01–1.31) higher prevalence of continuous work between year 2 and year 4 post-diagnosis in the multivariable model.

Similar results were observed in the population under age 60 at BC diagnosis (Suppl Table 4), with a 1.19 (1.05–1.35) higher prevalence of continuous work between year 2 and year 4 post-diagnosis among self-employed women compared to employees. In addition, compared to employees, higher ratios of RTW were observed among white-collar self-employed women 2 and 4 years after BC diagnosis, whereas no clear difference was reported for blue collar self-employed women. Results were similar in complete case analyses (Suppl Tables 5a and 5b).

## Discussion

In the three populations investigated, about 8% of women were self-employed. Return to work was marginally higher among the self-employed compared to employees at 2 and 4 years after diagnosis. This was limited to white collar self-employed women. Two years after diagnosis, self-employed women were more likely to maintain continuous employment until year 4 post-diagnosis compared to employees. These associations were similar although slightly more pronounced in the sensitivity analyses among women aged < 60 years at BC diagnosis.

The literature on self-employment and work-related outcomes after BC shows mixed results. Some studies measuring RTW at a single post-BC diagnosis time point found differing results. Bouknight^17^, found no difference on RTW between the self-employed and employees at 12 and 18 months post- BC diagnosis. However, other studies reported that self-employed women were more likely to work one or three years after BC than employees^18,19^. Other studies, like ours, have also assessed sustained RTW. Although studies like Torp et al.^12^ suggested self-employed workers took less time off after cancer than their employees counterparts, few studies measured RTW to account for work trajectories over time and interruptions following RTW^27^. In this study, we observed that once self-employed women are back to work, they are more likely to work continuously whereas employees tend to experience interruptions. This highlights the importance of understanding RTW as a process and not a one-time event that needs to be successfully tackled^28^. This was suggested in previous research where compared to employees, self-employed workers tended to leave employment less often^4^ and experienced more job changes following a cancer diagnosis^12,15,29^. Future research should explore these aspects.

In our study, we have evaluated the independent impact of being self-employed on RTW, measured both at a single time point RTW and as sustained RTW. In particular, we adjusted for potential confounding and mediating factors of this association, namely clinical and health-related factors, as well as work-related factors. Notably, the association between self-employment and RTW remained stable after adjustment, highlighting the intrinsic importance of job status in the process of returning to work. Although residual confounding cannot be ruled out as information on several potential confounding factors was lacking (e.g. distance between domicile and workplace), several employment status specificities could explain our results. First, financial factors are likely to exacerbate the differences in how the self-employed and employees react after a cancer diagnosis^12,15^. In many countries as in France, self-employed workers have a poorer social coverage and receive less financial compensation in case of sick leave^12,16^. In addition, sick leave could lead to the bankruptcy of their professional activity. The financial consequences of cancer are thus likely to be more pronounced among self-employed workers^30^. The decrease in salary after cancer diagnosis is more important for the self-employed than for employees^31^. Additionally, self-employed workers do not have superiors or organizations to rely on for assistance and support and they generally perceive the institutional support as not useful, because it is not adapted to their situation^5,15^. In contrast, self-employment allows for some freedom and autonomy in terms of work pace and schedules, allowing self-employed workers to maintain their employment and shift to less tiring tasks during certain periods^32^. Also, self-employed workers experience greater job satisfaction and may be more motivated to return to work compared to employees^4,20^.

Self-employment is heterogeneous in terms of working conditions, grouping non-manual and manual jobs^15^. It is likely that the physically demanding jobs make RTW more challenging among the self-employed than in the general population^2^. Unfortunately, we did not have this information in our data, but we believe education is a good proxy to explore this issue. Although we acknowledge this is not perfect as some physically demanding jobs (e.g. nurse) are in the high educated group, our results suggested higher RTW among white-collar self-employed survivors but not among blue-collar self-employed survivors compared to employee survivors, but these differences strongly decreased in multivariable analyses, pointing to the role of health- and work-related factors in these differences. However, we acknowledge the risk of a possible misclassification bias, which we hypothesize may lead us to an underestimation of the inequalities. Using the alternative cut-point of high school plus two years we observe no considerable differences between our results and the new results (Supplementary Table 6). A difference we observe is that white collar self-employees are more likely to return to work (PR = 1.13, 95%CI = 1.07–1.20) with the alternative threshold. However, this categorization is not free from criticism, as the group labelled ‘blue collar self-employed’ also encompasses a substantial proportion of self-employed women with high qualified job. Of note, while similar results were observed when the analyses were conducted among the larger sample of women aged up to 60 at diagnosis our results may not be generalizable to older individuals. Future studies should aim at studying the patterns of retirement among older self-employed survivors.

Our analysis is based on data from the CANTO cohort, which presents several methodological strengths and weaknesses. The prospective data collection allowed us to consider the temporality between covariates and RTW. However, as for any cohort, our population is not free from attrition bias regarding those lost to follow-up as well as selection bias regarding non-responders. However, based on the information from Supplementary Tables 2 and 3, individuals with a higher baseline risk of non-RTW were less likely to remain in the study cohorts, which may have led to a modest underestimation of the true associations. Our analyses considered various covariates such as sociodemographic, professional, clinical, and quality of life variables whereas the literature usually has limited detailed information on both clinical and sociodemographic data. Furthermore, we measured quality of life using validated PROMs, which are better correlated with patients’ health status than estimates made by physicians^33^. The large size of the CANTO cohort covering the metropolitan French territory allowed us to explore the heterogeneity among self-employed women. Despite its multicenter design, CANTO is based on large healthcare centers, which may limit its representativeness. However, we counted 8.1% of self-employed women in our data, a ratio similar to national estimates of 7.9% in 2018^21^. In addition, in France two years after diagnosis, the probability of being employed was increased by 7% point among the self-employed compared to employees whereas we observe a 5% point difference (85% vs. 80% respectively, see Table 2)^4^. Furthermore, in our study we did not study the impact of family on RTW and sustained-RTW. These could act as potential confounders. In addition, although in the present study we are able to quantify RTW, a relevant next question is the quality of such RTW. At the present time, we do not dispose of this information or similar to provide any additional analyses including the quality of the RTW. Future studies should investigate these issues.

Our analysis contributes to the literature on the role of employment status on RTW after cancer by investigating detailed work trajectories. While our results reveal no significant difference in RTW at 2 years and 4 years post-BC diagnosis, we found that self-employed workers return to work in a more sustained way than employees even after accounting for clinical and health-related factors. Because this is likely due to necessity rather than ability, it is crucial to develop measures addressing the unique challenges faced by self-employed individuals. These measures should particularly aim to support them in maintaining their business operations during periods of leave thereby helping them balance the demands of their business with their health needs. Policymakers should explore flexible sick leave models, tailored psychosocial support, and financial aid specifically designed for self-employed cancer survivors.

**Table 1:** Characteristics of women under age 57 at diagnosis in the three study populations. Imputed data. CANTO, France.

Characteristics	2-year RTW cohort			4-year RTW cohort			Continuous work cohort		
	Self-employed (*n* = 237)^1^	Employees (*n* = 2941)^1^	*p* ^2^	Self-employed (*n* = 171)^1^	Employees (*n* = 2158)^1^	*p* ^2^	Self-employed (*n* = 133)^1^	Employees (*n* = 1550)^1^	*p* ^2^
Clinical factors at diagnosis									
Stage			0.06			0.26			0.07
Stage I	51	43		50	44		57	47	
Stage II	40	46		39	46		36	44	
Stage III	9	11		11	10		7	9	
Charlson comorbidity index at diagnosis ≥ 1	13	13	0.94	11	13	0.49	10	11	0.85
Having > = 3 additional comorbid conditions at diagnosis ^3^	20	21	0.70	20	23	0.45	19	22	0.57
Received radiotherapy	90	93	0.20	93	93	1	94	93	0.90
Received anti Her2 therapy	16	15	0.63	18	15	0.49	17	14	0.41
Received chemotherapy	58	64	0.07	63	64	0.80	59	62	0.57
Received hormonal therapy	84	83	0.69	83	82	0.92	83	84	1
Had mastectomy (vs. conservative surgery)	29	30	0.71	27	30	0.50	25	27	0.63
Had lymph nodes dissection (vs. None or sentinel)	39	43	0.34	42	42	0.98	40	40	1
Sociodemographic and work-related variables at diagnosis									
Age, years			0.02			0.03			0.02
18–39	9	13		6	13		6	14	
40–49	43	47		47	47		46	47	
50–56	48	40		47	40		48	39	
Having dependent children	67	69	0.51	66	69	0.49	66	70	0.47
Having a partner	86	84	0.49	85	84	0.71	86	85	0.87
Household income per consumption unit (€)			0.30			0.18			0.17
< 1500	29	27		31	26		29	23	
[1500 ; 2000[	19	24		19	24		20	23	
[2000 ; 3000[	26	27		24	28		22	29	
≥ 3000	26	22		26	22		29	25	
Part-time work (vs. full-time work)	23	23	0.75	22	23	0.72	22	22	0.92
Work-life imbalance			< 0.001			< 0.001			< 0.001
Equal importance to personal and professional life	50	50		51	50		55	52	
Personal life is more important	31	41		30	40		26	39	
Professional life is more important	19	9		19	10		19	9	
Quality of life^4^									
Severe fatigue	34	42	0.02	32	40	0.07	29	37	0.06
Distress			0.56			0.33			0.26
Non case	64	61		62	59		67	64	
Doubtful case	23	24		21	26		19	25	
Case	13	15		17	15		14	11	
Physical functioning	27	33	0.07	26	29	0.39	19	23	0.30

^1^%.

^2^Pearson’s Chi-squared test; Wilcoxon rank sum test.

^3^Medical history not taken into account by the Charlson index covering neurological, cardiovascular, respiratory, gastrointestinal, renal, hepatobiliary, endocrine, musculoskeletal, urogenital, hematology, dermatology and psychiatry.

^4^Quality of life was self-reported 1 year after diagnosis when studying RTW 2 years after diagnosis. Quality of life was self-reported 2 years after diagnosis when studying RTW 4 years after diagnosis and continuous work between year 2 and year 4 post-diagnosis Fatigue and physical functioning were measured using the QLQ-C30 questionnaire, distress was measured using the HADS questionnaire. Severe fatigue was defined using the score of 39. Distress was categorized using the following thresholds: noncase [0–14], doubtful [15–22], case [over 23].

**Table 2:** Association between job status and work-related outcomes for women under 57. Univariable and multivariable analysis on imputed data. Poisson regression with robust variance. CANTO, France.

	Prevalence	Univariable model PR (95% CI)	Multivariable model* PR (95% CI)
RTW 2 years after diagnosis			
Employees	80	1	1
Self-employed	85	1.06 (1.01 ; 1.12)	1.04 (0.98 ; 1.10)
Employees	80	1	1
Blue collar self-employed	79	0.99 (0.89 ; 1.09)	1.00 (0.90 ; 1.11)
White collar self-employed	89	1.11 (1.05 ; 1.18)	1.05 (1.00 ; 1.12)
RTW 4 years after diagnosis			
Employees	81	1	1
Self-employed	86	1.06 (0.99 ; 1.12)	1.05 (0.99 ; 1.12)
Employees	81	1	1
Blue collar self-employed	79	0.97 (0.86 ; 1.10)	1.04 (0.92 ; 1.18)
White collar self-employed	90	1.11 (1.04 ; 1.19)	1.06 (0.99 ; 1.13)
Continuous work between year 2 and year 4 post-diagnosis among women who returned to work 2 years after diagnosis			
Employees	57	1	1
Self-employed	67	1.18 (1.04 ; 1.34)	1.15 (1.01 ; 1.31)

*PR* Prevalence Ratio, *95% CI* 95% Confidence Interval.

*Adjusted model on socioeconomic factors at diagnosis (age, presence of a partner, presence of dependent children, income per consumption unit, weekly working hours, work-life imbalance), clinical factors (stage at diagnosis, Charlson comorbidity index at inclusion, additional comorbid conditions at inclusion, radiotherapy, anti Her2 therapy, chemotherapy, hormonal therapy, breast surgery, lymph nodes dissection) and quality of life (fatigue, distress, physical functioning). We used quality of life self-reported 1 year after diagnosis when investigating RTW 2 years after diagnosis. We used quality of life self-reported 2 years after diagnosis when investigating RTW 4 years after diagnosis and continuous work between year 2 and year 4 post-diagnosis. Fatigue and physical functioning were measured using the QLQ-C30 questionnaire, distress was measured using the HADS questionnaire.

## Supplementary Information

Below is the link to the electronic supplementary material.

Supplementary Material 1

## Data Availability

Deidentified participant data that support the findings of this study are available from UNICANCER upon reasonable request. Data access requires approval by the CANTO scientific committee and the signing of a data access agreement. Requests should be directed to canto@unicancer.fr.
