# Supplementary material for: Return to work among self-employed breast cancer survivors from the CANTO cohort
Source: Sci Rep. 2026 Mar 12;16:13195. doi: 10.1038/s41598-026-41157-3 (PMC13103402; doi:10.1038/s41598-026-41157-3)
Supplement: Supplementary file 1 — Supplementary Material 1 [file 41598_2026_41157_MOESM1_ESM.docx]

Suppl Table 1a. Characteristics of women under age 57 at diagnosis in the study population that returned to work at T2. Data not imputed.

| **Variable** | **Value** | **N** | **Percent** |
| --- | --- | --- | --- |
| Having >= 3 additional comorbid conditions at diagnosis | NA | 198 | 6,2 |
|  | 0 | 2338 | 73,6 |
|  | 1 | 642 | 20,2 |
| Had lymph nodes dissection (vs None or sentinel) | Dissection | 1348 | 42,4 |
| Charlson comorbidity index at diagnosis ≥ 1 | NA | 233 | 7,3 |
|  | 0 | 2552 | 80,3 |
|  | >=1 | 393 | 12,4 |
| Chemotherapy | 0 | 1163 | 36,6 |
|  | 1 | 2015 | 63,4 |
| Distress at T1 | NA | 232 | 7,3 |
|  | a: Normal | 1796 | 56,5 |
|  | b: Borderline | 702 | 22,1 |
|  | c: Case | 448 | 14,1 |
| Anti-Her2 therapy | 0 | 2709 | 85,2 |
|  | 1 | 469 | 14,8 |
| Hormone therapy | 0 | 546 | 17,2 |
|  | 1 | 2632 | 82,8 |
| Type of contract | Self-employed | 237 | 7,5 |
|  | Salariees | 2941 | 92,5 |
| Radiotherapy | NA | 1 | 0 |
|  | 0 | 235 | 7,4 |
|  | 1 | 2942 | 92,6 |
| Type of surgery | Conservative surgery | 2212 | 69,6 |
|  | Mastectomy | 966 | 30,4 |
| Age | 18-39 | 409 | 12,9 |
|  | 40-49 | 1482 | 46,6 |
|  | 50-56 | 1287 | 40,5 |
| Physical functioning at T1 | NA | 224 | 7 |
|  | 0 | 1984 | 62,4 |
|  | 1 | 970 | 30,5 |
| Severe Fatigue at T1 | NA | 228 | 7,2 |
|  | 0 | 1728 | 54,4 |
|  | 1 | 1222 | 38,5 |
| Stage at diagnosis | NA | 34 | 1,1 |
|  | I | 1392 | 43,8 |
|  | II | 1418 | 44,6 |
|  | III | 334 | 10,5 |

**Suppl Table 1b.** Characteristics of women under age 57 at diagnosis in the study population that returned to work at T4. Data not imputed.

| **Variable** | **Value** | **N** | **Percent** |
| --- | --- | --- | --- |
| Having >= 3 additional comorbid conditions at diagnosis | NA | 133 | 5,7 |
|  | 0 | 1685 | 72,3 |
|  | 1 | 511 | 21,9 |
| Had lymph nodes dissection (vs None or sentinel) | Dissection | 972 | 41,7 |
| Charlson comorbidity index at diagnosis ≥ 1 | NA | 160 | 6,9 |
|  | 0 | 1891 | 81,2 |
|  | >=1 | 278 | 11,9 |
| Chemotherapy | 0 | 844 | 36,2 |
|  | 1 | 1485 | 63,8 |
| Distress at T1 | NA | 242 | 10,4 |
|  | a: Normal | 1247 | 53,5 |
|  | b: Borderline | 531 | 22,8 |
|  | c: Case | 309 | 13,3 |
| Anti-Her2 therapy | 0 | 1970 | 84,6 |
|  | 1 | 359 | 15,4 |
| Hormone therapy | 0 | 408 | 17,5 |
|  | 1 | 1921 | 82,5 |
| Type of contract | Self-employed | 171 | 7,3 |
|  | Salariees | 2158 | 92,7 |
| Radiotherapy | 0 | 170 | 7,3 |
|  | 1 | 2159 | 92,7 |
| Type of surgery | Conservative surgery | 1643 | 70,5 |
|  | Mastectomy | 686 | 29,5 |
| Age | 18-39 | 295 | 12,7 |
|  | 40-49 | 1084 | 46,5 |
|  | 50-56 | 950 | 40,8 |
| Physical functioning at T1 | NA | 219 | 9,4 |
|  | 0 | 1488 | 63,9 |
|  | 1 | 622 | 26,7 |
| Severe Fatigue at T1 | NA | 225 | 9,7 |
|  | 0 | 1265 | 54,3 |
|  | 1 | 839 | 36 |
| Stage at diagnosis | NA | 31 | 1,3 |
|  | I | 1037 | 44,5 |
|  | II | 1039 | 44,6 |
|  | III | 222 | 9,5 |

**Suppl Table 1c.** Characteristics of women under age 57 at diagnosis in the study population that continued to work between T2 and T4. Data not imputed.

| **Variable** | **Value** | **N** | **Percent** |
| --- | --- | --- | --- |
| Having >= 3 additional comorbid conditions at diagnosis | NA | 133 | 5,7 |
|  | 0 | 1685 | 72,3 |
|  | 1 | 511 | 21,9 |
| Had lymph nodes dissection (vs None or sentinel) | Dissection | 972 | 41,7 |
| Charlson comorbidity index at diagnosis ≥ 1 | NA | 160 | 6,9 |
|  | 0 | 1891 | 81,2 |
|  | >=1 | 278 | 11,9 |
| Chemotherapy | 0 | 844 | 36,2 |
|  | 1 | 1485 | 63,8 |
| Distress at T1 | NA | 242 | 10,4 |
|  | a: Normal | 1247 | 53,5 |
|  | b: Borderline | 531 | 22,8 |
|  | c: Case | 309 | 13,3 |
| Anti-Her2 therapy | 0 | 1970 | 84,6 |
|  | 1 | 359 | 15,4 |
| Hormone therapy | 0 | 408 | 17,5 |
|  | 1 | 1921 | 82,5 |
| Type of contract | Self-employed | 171 | 7,3 |
|  | Salariees | 2158 | 92,7 |
| Radiotherapy | 0 | 170 | 7,3 |
|  | 1 | 2159 | 92,7 |
| Type of surgery | Conservative surgery | 1643 | 70,5 |
|  | Mastectomy | 686 | 29,5 |
| Age | 18-39 | 295 | 12,7 |
|  | 40-49 | 1084 | 46,5 |
|  | 50-56 | 950 | 40,8 |
| Physical functioning at T1 | NA | 219 | 9,4 |
|  | 0 | 1488 | 63,9 |
|  | 1 | 622 | 26,7 |
| Severe Fatigue at T1 | NA | 225 | 9,7 |
|  | 0 | 1265 | 54,3 |
|  | 1 | 839 | 36 |
| Stage at diagnosis | NA | 31 | 1,3 |
|  | STADE I | 1037 | 44,5 |
|  | STADE II | 1039 | 44,6 |
|  | STADE III | 222 | 9,5 |

**Suppl table 2.** Characteristics of non-respondents two years after BC diagnosis. CANTO cohort

|  | Lost to follow-up, consent withdrawn, relapse, or death |  |  | No reply to the questionnaire | |
| --- | --- | --- | --- | --- | --- |
|  | Self-employed  N = 33 | Employees  N = 348 |  | Self-employed  N = 93 | Employees  N = 829 |
| **Age** |  |  |  |  |  |
| 18-39 | 8 (24%) | 51 (15%) |  | 9 (9.7%) | 140 (17%) |
| 40-49 | 14 (42%) | 148 (43%) |  | 46 (49%) | 413 (50%) |
| 50-56 | 11 (33%) | 149 (43%) |  | 38 (41%) | 276 (33%) |
| **Education** |  |  |  |  |  |
| Higher than high school | 16 (52%) | 146 (44%) |  | 54 (58%) | 418 (51%) |
| High school or less | 15 (48%) | 187 (56%) |  | 39 (42%) | 394 (49%) |
| **Stage at diagnosis** |  |  |  |  |  |
| Stage I | 10 (31%) | 127 (38%) |  | 41 (44%) | 359 (44%) |
| Stage II | 21 (66%) | 147 (44%) |  | 43 (46%) | 382 (47%) |
| Stage III | 1 (3.1%) | 58 (17%) |  | 9 (9.7%) | 72 (8.9%) |
| **Charlson comorbidity index at diagnosis** |  |  |  |  |  |
| >=1 | 3 (9.7%) | 46 (14%) |  | 10 (12%) | 118 (16%) |
| 0 | 28 (90%) | 272 (86%) |  | 73 (88%) | 607 (84%) |
| **Radiotherapy** |  |  |  |  |  |
| No | 6 (18%) | 34 (10%) |  | 12 (13%) | 63 (7.6%) |
| Yes | 27 (82%) | 305 (90%) |  | 81 (87%) | 766 (92%) |
| **Anti Her2 therapy** |  |  |  |  |  |
| No | 32 (97%) | 292 (86%) |  | 79 (85%) | 700 (84%) |
| Yes | 1 (3.0%) | 49 (14%) |  | 14 (15%) | 129 (16%) |
| **Chemotherapy** |  |  |  |  |  |
| No | 11 (33%) | 117 (34%) |  | 42 (45%) | 300 (36%) |
| Yes | 22 (67%) | 225 (66%) |  | 51 (55%) | 529 (64%) |
| **Hormonal therapy** |  |  |  |  |  |
| No | 17 (52%) | 100 (29%) |  | 17 (18%) | 157 (19%) |
| Yes | 16 (48%) | 240 (71%) |  | 76 (82%) | 672 (81%) |
| **Breast surgery** |  |  |  |  |  |
| Conservative surgery | 24 (73%) | 219 (64%) |  | 55 (59%) | 581 (70%) |
| Mastectomy | 9 (27%) | 125 (36%) |  | 38 (41%) | 248 (30%) |
| **Lymph nodes dissection** |  |  |  |  |  |
| Dissection | 13 (39%) | 165 (48%) |  | 34 (37%) | 330 (40%) |
| None or sentinel | 20 (61%) | 179 (52%) |  | 59 (63%) | 499 (60%) |

**Suppl table 3.** Characteristics of non-respondents four years after BC diagnosis. CANTO cohort

|  | Lost to follow-up, consent withdrawn,  relapse, or death | | No reply  to the questionnaire | |
| --- | --- | --- | --- | --- |
|  | Self-employed  N = 79 | Employees  N = 746 | Self-employed  N = 133 | Employees  N = 1,214 |
| **Age** |  |  |  |  |
| 18-39 | 15 (19%) | 116 (16%) | 11 (9.7%) | 180 (15%) |
| 40-49 | 36 (46%) | 337 (45%) | 47 (42%) | 599 (49%) |
| 50-56 | 28 (35%) | 293 (39%) | 55 (49%) | 435 (36%) |
| **Education** |  |  |  |  |
| Higher than high school | 36 (47%) | 331 (46%) | 70 (63%) | 633 (53%) |
| High school or less | 41 (53%) | 392 (54%) | 42 (38%) | 563 (47%) |
| **Stage at diagnosis** |  |  |  |  |
| Stage I | 28 (36%) | 280 (39%) | 59 (53%) | 525 (44%) |
| Stage II | 42 (54%) | 323 (44%) | 48 (43%) | 559 (47%) |
| Stage III | 8 (10%) | 124 (17%) | 5 (4.5%) | 115 (9.6%) |
| **Charlson comorbidity index at diagnosis** |  |  |  |  |
| >=1 | 10 (13%) | 94 (14%) | 15 (15%) | 173 (16%) |
| 0 | 65 (87%) | 596 (86%) | 88 (85%) | 892 (84%) |
| **Radiotherapy** |  |  |  |  |
| No | 13 (16%) | 58 (7.9%) | 16 (14%) | 93 (7.7%) |
| Yes | 66 (84%) | 679 (92%) | 97 (86%) | 1,120 (92%) |
| **Anti Her2 therapy** |  |  |  |  |
| No | 72 (91%) | 635 (86%) | 97 (86%) | 1,038 (86%) |
| Yes | 7 (8.9%) | 104 (14%) | 16 (14%) | 176 (14%) |
| **Chemotherapy** |  |  |  |  |
| No | 28 (35%) | 253 (34%) | 61 (54%) | 447 (37%) |
| Yes | 51 (65%) | 487 (66%) | 52 (46%) | 767 (63%) |
| **Hormonal therapy** |  |  |  |  |
| No | 24 (30%) | 189 (26%) | 19 (17%) | 197 (16%) |
| Yes | 55 (70%) | 549 (74%) | 94 (83%) | 1,017 (84%) |
| **Breast surgery** |  |  |  |  |
| Conservative surgery | 52 (66%) | 482 (65%) | 70 (62%) | 844 (70%) |
| Mastectomy | 27 (34%) | 260 (35%) | 43 (38%) | 370 (30%) |
| **Lymph nodes dissection** |  |  |  |  |
| Dissection | 33 (42%) | 366 (49%) | 35 (31%) | 484 (40%) |
| None or sentinel | 46 (58%) | 376 (51%) | 78 (69%) | 730 (60%) |

| **Suppl Table 4.** Association between job status (Self-employed/ Employees) and work-related outcomes for women under 60. Univariable and multivariable analysis on imputed data. Poisson regression with robust variance. CANTO, France, 2012-2018. | | | | |
| --- | --- | --- | --- | --- |
|  | Prevalence | Univariable model  PR (95% CI) |  | Multivariable model*  PR (95% CI) |
| ***RTW 2 years after diagnosis*** | | | | |
| Employees  Self-employed | 78  83 | 1  1.07 (1.01 ; 1.13) |  | 1  1.04 (0.98 ; 1.10) |
| Employees  Blue collar self-employed  White collar self-employed | 78  76  88 | 1  0.98 (0.88 ; 1.09)  1.13 (1.07 ; 1.20) |  | 1  0.98 (0.88 ; 1.10)  1.07 (1.01 ; 1.14) |
| ***RTW 4 years after diagnosis*** | | | | |
| Employees  Self-employed | 75  81 | 1  1.08 (1.00 ; 1.16) |  | 1  1.08 (1.01 ; 1.16) |
| Employees  Blue collar self-employed  White collar self-employed | 75  71  87 | 1  0.95 (0.82 ; 1.10)  1.16 (1.08 ; 1.25) |  | 1  1.03 (0.90 ; 1.19)  1.10 (1.03 ; 1.18) |
| ***Continuous work*** ***between year 2 and year 4 post-diagnosis*** | | | | |
| Employees  Self-employed | 55  65 | 1  1.20 (1.06 ; 1.33) |  | 1  1.19 (1.05 ; 1.35) |
| PR: Prevalence Ratio; 95% CI: 95 % Confidence Interval  *Adjusted model on socioeconomic factors at diagnosis (age, presence of a partner, presence of dependent children, income per consumption unit, weekly working hours, work-life imbalance), clinical factors (stage at diagnosis, Charlson comorbidity index at inclusion, additional comorbid conditions at inclusion, radiotherapy, anti Her2 therapy, chemotherapy, hormonal therapy, breast surgery, lymph nodes dissection) and quality of life (fatigue, distress, physical functioning). We used quality of life self-reported 1 year after diagnosis when investigating RTW 2 years after diagnosis. We used quality of life self-reported 2 years after diagnosis when investigating RTW 4 years after diagnosis and continuous work between year 2 and year 4 post-diagnosis. Fatigue and physical functioning were measured using the QLQ-C30 questionnaire, distress was measured using the HADS questionnaire. | | | | |

| Suppl Table 5a: Association between job status and work-related outcomes for women under 57. Univariate and multivariate analysis on complete case. Poisson regression with robust variance. CANTO, France. | | | | |
| --- | --- | --- | --- | --- |
|  | Prevalence | Univariate model  PR (95% CI) |  | Multivariate model*  PR (95% CI) |
| ***RTW 2 years after diagnosis*** | | | | |
| Salaried  Self-employed | 80  85 | 1  1.06 (1.01 ; 1.12) |  | 1  1.01 (0.94 ; 1.09) |
| Salaried  Blue collar self-employed  White collar self-employed | 80  79  89 | 1  0.99 (0.89 ; 1.09)  1.11 (1.05 ; 1.18) |  | 1  0.94 (0.81 ; 1.08)  1.05 (0.98 ; 1.14) |
| ***RTW 4 years after diagnosis*** | | | | |
| Salaried  Self-employed | 81  86 | 1  1.06 (0.99 ; 1.13) |  | 1  1.04 (0.96 ; 1.13) |
| Salaried  Blue collar self-employed  White collar self-employed | 81  79  90 | 1  0.97 (0.86 ; 1.10)  1.11 (1.04 ; 1.19) |  | 1  0.92 (0.76 ; 1.12)  1.10 (1.02 ; 1.19) |
| ***Continuous work between year 2 and year 4 post-diagnosis among women who returned to work 2 years after diagnosis*** | | | | |
| Salaried  Self-employed | 57  67 | 1  1.18 (1.04 ; 1.34) |  | 1  1.13 (0.96 ; 1.32) |
| PR: Prevalence Ratio; 95% CI: 95 % Confidence Interval  *Adjusted model on socioeconomic factors at diagnosis (age, presence of a partner, presence of dependent children, income per consumption unit, weekly working hours, work-life imbalance), clinical factors (stage at diagnosis, Charlson comorbidity index at inclusion, additional comorbid conditions at inclusion, radiotherapy, anti Her2 therapy, chemotherapy, hormonal therapy, breast surgery, lymph nodes dissection) and quality of life (fatigue, distress, physical functioning). We used quality of life self-reported 1 year after diagnosis when investigating RTW 2 years after diagnosis. We used quality of life self-reported 2 years after diagnosis when investigating RTW 4 years after diagnosis and continuous work between year 2 and year 4 post-diagnosis. Fatigue and physical functioning were measured using the QLQ-C30 questionnaire, distress was measured using the HADS questionnaire. | | | | |

| Suppl Table 5b. Association between job status (Self-employed/ Salaried) and work-related outcomes for women under 60. Univariate and multivariate analysis on complete case. Poisson regression with robust variance. CANTO, France, 2012-2018. | | | | |
| --- | --- | --- | --- | --- |
|  | Prevalence | Univariate model  PR (95% CI) |  | Multivariate model*  PR (95% CI) |
| ***RTW 2 years after diagnosis*** | | | | |
| Salaried  Self-employed | 78  83 | 1  1.07 (1.01 ; 1.13) |  | 1  1.04 (0.96 ; 1.11) |
| Salaried  Blue collar self-employed  White collar self-employed | 78  76  88 | 1  0.98 (0.88 ; 1.09)  1.13 (1.07 ; 1.20) |  | 1  0.93 (0.81 ; 1.08)  1.06 (0.99 ; 1.14) |
| ***RTW 4 years after diagnosis*** | | | | |
| Salaried  Self-employed | 75  81 | 1  1.08 (1.00** ; 1.16) |  | 1  1.09 (1.00** ; 1.19) |
| Salaried  Blue collar self-employed  White collar self-employed | 75  71  87 | 1  0.95 (0.82 ; 1.10)  1.16 (1.08 ; 1.25) |  | 1  0.95 (0.77 ; 1.17)  1.14 (1,04 ; 1.24) |
| ***Continuous work*** ***between year 2 and year 4 post-diagnosis*** | | | | |
| Salaried  Self-employed | 55  65 | 1  1.20 (1.06 ; 1.36) |  | 1  1.15 (0.99 ; 1.35) |
| PR: Prevalence Ratio; 95% CI: 95 % Confidence Interval  *Adjusted model on socioeconomic factors at diagnosis (age, presence of a partner, presence of dependent children, income per consumption unit, weekly working hours, work-life imbalance), clinical factors (stage at diagnosis, Charlson comorbidity index at inclusion, additional comorbid conditions at inclusion, radiotherapy, anti Her2 therapy, chemotherapy, hormonal therapy, breast surgery, lymph nodes dissection) and quality of life (fatigue, distress, physical functioning). We used quality of life self-reported 1 year after diagnosis when investigating RTW 2 years after diagnosis. We used quality of life self-reported 2 years after diagnosis when investigating RTW 4 years after diagnosis and continuous work between year 2 and year 4 post-diagnosis. Fatigue and physical functioning were measured using the QLQ-C30 questionnaire, distress was measured using the HADS questionnaire.  ** Confidence interval does not include 1. | | | | |
